# The Astaxanthin Aggregation Pattern Greatly Influences Its Antioxidant Activity: A Comparative Study in Caco-2 Cells

**DOI:** 10.3390/antiox9020126

**Published:** 2020-02-02

**Authors:** Mingqin Dai, Chunjun Li, Zhao Yang, Zhe Sui, Jing Li, Ping Dong, Xingguo Liang

**Affiliations:** 1 College of food Science and Engineering, Ocean University of China, Qingdao 266003, China; daimingqin123@163.com (M.D.); lichunjun1@126.com (C.L.); 18753858362@163.com (Z.Y.); suizhe@ouc.edu.cn (Z.S.); liangxg@ouc.edu.cn (X.L.); 2 Laboratory for Marine Drugs and Bioproducts, Qingdao National Laboratory for Marine Science and Technology, Qingdao 266235, China

**Keywords:** carotenoids, colloidal astaxanthin, H-aggregate, J-aggregate, antioxidation

## Abstract

Astaxanthin is an excellent antioxidant that can form unstable aggregates in biological or artificial systems. The changes of astaxanthin properties caused by molecular aggregation have gained much attention recently. Here, water-dispersible astaxanthin H- and J-aggregates were fabricated and stabilized by a natural DNA/chitosan nanocomplex (respectively noted as H-ADC and J-ADC), as evidenced by ultraviolet and visible spectrophotometry, Fourier transform infrared spectroscopy, and Raman spectroscopy. Compared with J-ADC, H-ADC with equivalent astaxanthin loading capacity and encapsulation efficiency showed smaller particle size and similar zeta potential. To explore the antioxidant differences between astaxanthin H- and J-aggregates, H-ADC and J-ADC were subjected to H_2_O_2_-pretreated Caco-2 cells. Compared with astaxanthin monomers and J-aggregates, H-aggregates showed a better cytoprotective effect by promoting scavenging of intracellular reactive oxygen species. Furthermore, in vitro 1,1-diphenyl-2-picrylhydrazyl and hydroxyl free radical scavenging studies confirmed a higher efficiency of H-aggregates than J-aggregates or astaxanthin monomers. These findings give inspiration to the precise design of carotenoid aggregates for efficient utilization.

## 1. Introduction

There is overwhelming evidence that carotenoids play an important role in antioxidant activity. Astaxanthin (3,3′-dihydroxy-β,β’-carotene-4,4′-dione, AST) is a chiral xanthophyll that plays essential photochemical and physiological roles in animals, plants and microorganisms [1]. This carotenoid pigment has been found in most crustaceans, microalgae, and microorganisms [2]. Compared to synthetic AST, natural AST extracted from bioproducts, such as enzymatic hydrolysis of shrimp waste, supercritical fluid extraction from *Parapeneus longirostris*, etc. [3,4], showed better antioxidant activity. Despite its poor stability and water solubility, natural AST has gained lots of attention due to its excellent antioxidant, anti-inflammatory, immunomodulatory, photoprotection, neuroprotection and tumor inhibition activities [5]. To improve the stability and the bioavailability of AST, many strategies have been employed to encapsulate AST. For example, Machado et al. [6] used supercritical fluids to enhance the water dispersibility of AST, and they obtained an AST colloidal system with a particle size of 265 nm. However, the AST encapsulation efficiency was only 26.1% with a loading capacity of 7.5%. By using an emulsification-solvent evaporation method, Zanoni et al. [7] prepared AST/whey protein nanoparticles with an encapsulation efficiency as high as 96%. The nanoparticles showed a particle size of 130 nm with a negative zeta potential value of 30 mV. According to Ma’s report [8], particles with a positive surface charge were more favorable for cellular uptake compared to negatively-charged particles. To enhance the intracellular antioxidant activity of AST, Hu [9] and his coworkers synthesized AST-loaded PLGA nanoparticles for anti-photodamage on skin cells (HaCaT), and optimized the nanoparticles with a particle size of 154.4 nm, zeta potential of 22.07 mV, encapsulation rate of 96.42% and AST loading capacity of 7.19%. In our previous work, natural DNA/chitosan co-assemblies were employed as novel nanocarriers for oral delivery of AST. The prepared nanoparticles with a zeta potential of 35.3 mV and particle size of 211 nm showed excellent antioxidant activity on H_2_O_2_-damaged Caco-2 cells [10]. Regrettably, the aggregation form of AST in the above-mentioned colloidal systems was not clear or beyond control.

Biomolecular aggregation is common in nature and important for the proper functioning of biological systems [11,12]. Aggregation changes the shape and physical properties of hydrophobic biomolecules, thereby affecting their bioavailability and functions in subcellular structures [13,14]. Taking carotenoids as an example, the aggregation form of carotenoids are widely found in living organisms with various significant functions, such as light-harvesting, photoprotection, and antioxidation [15,16]. Because aggregation of carotenoids occurs in various natural environments, the structure of the monomer and the local environment of the molecules would greatly affect the exact form of the aggregates [17,18]. It is essential to reveal the relationship between the aggregation forms of carotenoids and their biological function for better utilization of carotenoids.

As the predominant form of natural AST, all-trans-astaxanthin is found to have two different aggregation forms, H- and J-type aggregates, in addition to its monomer form. Although it is well accepted that the structure of AST determines its biological properties, few works could be found to reveal the relationship between AST aggregates and their physical and chemical properties due to the difficulties in obtaining stable AST aggregates [19,20]. Recently, we prepared two colloidal systems with differently aggregated AST to investigate the effect of dehydrated solvent on AST aggregation [21]. In this paper, AST H-aggregates and J-aggregates were precisely controlled and stably incorporated into DNA/chitosan nanoparticles by adjusting the feeding order of the polymers and ethanol content. Utilization of natural DNA and chitosan not only guarantees the safety of the AST colloidal system, but also minimizes the matrix differences of the formulations [22]. To provide a deeper understanding of how aggregation forms of AST influence its chemical and biological properties, AST H-aggregate-embedded DNA/chitosan nanoparticles (H-ADC) and AST J- aggregate-embedded DNA/chitosan nanoparticles (J-ADC) were characterized and their cellular antioxidant activity were evaluated using an oxidative stress damage cell model. In addition, DPPH• free radicals and hydroxyl free radicals were produced and used for scavenging rate analysis to directly show the free radicals scavenging capacity of H-ADC and J-ADC.

## 2. Materials and Methods

### 2.1. Materials

AST, 3-(4,5-dimethylthiazol-2-yl)-2,5-diphenyl-tetrazolium bromide (MTT), 2,7-dichlorodihydrofluorescein diacetate (DCFH-DA), fluorescein isothiocyanate (FITC) and salmon sperm DNA were obtained from Sigma (St. Louis, MO, USA). Chitosan (80 KDa, 91% deacetylation) was purchased from the Australia Hing Biological Technology Co, LTD (Zhejiang, China). All the other reagents and solvents were of analytical grade.

Caco-2 cells were obtained from the American Type Culture Collection (Rockville, MD, USA). Caco-2 cells were incubated in a humidified atmosphere containing 5% CO_2_ at 37 °C, which contained 90% Dulbecco’s modified Eagle medium (DMEM) (Gibco, Grand Island, NY, USA) and 10% fetal calf serum (Gibco, Grand Island, NY, USA).

### 2.2. Preparation and Characterization of AST Aggregate-Embedded Nano-Suspensions

#### 2.2.1. Preparation of AST Aggregate-Embedded Nano-Suspensions

Two kinds of AST aggregate-embedded nano-suspensions were prepared by two different methods. As for method 1, 20 mL of AST/ethanol solution (0.024 mg/mL) was first mixed with 160 mL of salmon sperm DNA solution (0.01 mg/mL) to maintain the ethanol/water volume ratio at 1:8. After 30 min of magnetic stirring, 80 mL of chitosan solution (0.04 mg/mL) was added to the solution for another 30 min of stirring. Ethanol was evaporated using a rotary evaporator (Buchi RE 121, Flavil, Switzerland) at 35 °C, and finally a type of AST aggregate-embedded DNA/chitosan nanoparticle (ADC) was obtained.

As for method 2, the amount of AST, DNA and chitosan remained the same as in method 1. The differences were: (1) the ethanol/water volume ratio was kept at 1:2; (2) the adding orders were changed by first mixing chitosan and AST, and then adding DNA. Finally, another type of ADC was obtained.

The products were stored at 20 °C under light protection for five days for further use. A typical tyndall light-scattering effect was observed by irradiating the freshly prepared aqueous suspension with a laser beam, confirming the colloidal stability.

#### 2.2.2. Ultraviolet and Visible Spectrophotometry (UV-Vis)

The UV-Vis spectra of different ADC nano-suspensions were recorded using a Shimadzu UV-1800 spectrophotometer according to Yang’s method [23]. The wavelength ranged from 350 nm to 700 nm with a slit of 1.0 nm and a high scan speed model. Each sample was measured three times. Compared with the AST monomers in ethanol solution, the ADC nano-suspension showed a blue-shifted absorption peak, named a H-ADC nano-suspension, while the nano-suspension showed a red-shifted absorption peak, named a J-ADC nano-suspension.

#### 2.2.3. Dynamic Light Scattering

The zeta potential, particle size, and polydispersity index (PdI) of H-ADC and J-ADC were surveyed by Zetasizer (3000HS, Malvern Instruments, Worcestershire, UK) according to Wang’s method [10].

#### 2.2.4. Transmission Electron Microscopy (TEM) 

The morphological structure of H-ADC and J-ADC were observed by TEM (JEM-2100 JEOL, Japan) according to Yang’s method [24]. A H-ADC and J-ADC nano-suspension was dripped onto a copper grid and observed after drying.

#### 2.2.5. Encapsulation Efficiency (EE) and Loading Capacity (LC)

The AST content in the nano-suspension was determined by HPLC (Chromer; HITACHI, Tokyo, Japan) according to Wang’s method [10]. The peak area vs. the AST concentration was linear in the range of the measured concentrations (*R*^2^ = 0.9975, *n* = 7). EE and LC was respectively calculated by Equations (1) and (2):

EE (%) = (amount of AST in the ADC/total amount of AST) × 100%(1)

LC (%) = (amount of AST in the ADC/total amount of the ADC) × 100%(2)

#### 2.2.6. Fourier Transform Infrared Spectroscopy (FT-IR)

FT-IR patterns of AST, H-ADC, and J-ADC were acquired using an ATR-Nicolet IS50 FT-IR spectrometer (Thermo Fisher Scientific, Waltham, MA, USA) referring to Zhao’s method [25]. OMNIC v8.2 software (Thermo-Nicolet, Wisconsin, USA) was used for data analysis.

#### 2.2.7. Raman Spectroscopic Investigation

Raman spectroscopic investigation of AST, H-ADC, J-ADC was acquired using a DXR Raman Microscope (Thermo Fisher Scientific, Co, LTD, Madison, WI, USA) referring to Subramanian’s method [26]. The Raman spectra laser was at a 532 nm wavelength, with each sample measured three times.

### 2.3. Cytoprotective Effect of H-ADC and J-ADC on Oxidative Stressed Caco-2 Cells

The ability of H-ADC or J-ADC to protect Caco-2 cells against H_2_O_2_-induced oxidative stress was evaluated according to the method of Wang [10]. Free AST, as well as a mixture of AST, DNA, and chitosan (noted as MIX) with the same content of ADC nano-suspension were used as positive controls. Different samples, including free AST, MIX, H-ADC or J-ADC nanoparticles (with an AST concentration of 8 μM) were used to evaluate their cryoprotective ability. The MTT assay was used to determine the cytoprotective effect of each sample [27]. Each sample was measured six times. The cytotoxicity of the samples was simultaneously studied for comparison. The protective effect of each sample on oxidative damage in Caco-2 cells was expressed as relative cell viability (%) by Equation (3):

Relative cell viability (%) = A_S_/A_C_ × 100% (3)

A_S_: = Absorbance of the tested sample; A_C_: = Absorbance of the negative control.

### 2.4. Reactive Oxygen Species (ROS) Scavenging Efficiency of ADC Nanoparticles in Caco-2 Cells

To determine the cell antioxidant effect of each antioxidant mentioned in Section 2.3, ROS scavenging efficiencies of H-ADC and J-ADC in Caco-2 cells were measured according to Wolfe’s method with a minor modification [28]. Briefly, free AST, MIX, H-ADC or J-ADC nanoparticles (with an AST concentration of 8 μM) were incubated with Caco-2 cells. The cells cultured in DMEM served as a negative control. After 24 h incubation, the cells were washed twice with PBS and cultured with DCFH-DA for 1 h. After removing the supernatants, the cells were washed with PBS, and then incubated with 100 μL H_2_O_2_ (5.8 mM) solution for 1 h. Finally, the fluorescent intensities were assayed using a fluorescence plate reader (λ_ex_ = 485 nm, λ_em_ = 535 nm). Each sample was measured six times. The general cellular antioxidant activity was expressed as ROS scavenging efficiency (%) by Equation (4):

ROS scavenging efficiency (%) = (1 − B_S_/B_C_) × 100% (4)

B_S_: The fluorescence intensities of the tested sample; B_C_: The fluorescence intensities of the negative control.

### 2.5. Free Radical Scavenging Ability 

To determine the free radical scavenging ability of each antioxidant mentioned in Section 2.3, 1,1-diphenyl-2-picrylhydrazyl free radicals (DPPH•) and hydroxyl radicals (HO•) were employed as typical free radicals. The DPPH• scavenging efficiency was assessed according to the method of Thaipong [29]. Briefly, 2 mL of the antioxidant solution with an AST concentration of 2, 5, 20 μM were added to 2 mL of 1,1-diphenyl-2-picrylhydrazyl/ethanol solution, respectively. After 30 min reaction, the mixture was centrifuged for 10 min at 2725 g/min. The absorbance of the supernatant was measured at 517 nm. Each sample was measured six times. The scavenging rate of DPPH• was calculated by Equation (5):

DPPH• scavenging rate (%) = [1 − (A_1_ − A_2_)/A_0_] × 100% (5)

A_0_: Absorption value of 2 mL anhydrous ethanol + 2 mL 1,1-diphenyl-2-picrylhydrazyl solution; A_1_: Absorption value of 2 mL sample solution + 2 mL 1,1-diphenyl-2-picrylhydrazyl solution; A_2_: Absorption value of 2 mL sample solution + 2 mL anhydrous ethanol.

The HO• scavenging ability of each antioxidant mentioned in Section 2.3 was evaluated by the method of Zhao [30]. Briefly, 0.5 mL of FeSO_4_ (1.5 mM) was reacted with 0.35 mL of H_2_O_2_ (6 mM) to generate HO•, 0.15 mL of sodium salicylate (20 mM) and 1 mL of antioxidant at different concentrations (2, 5 or 20 µM) were added to hydroxylate salicylate. After incubation for 1 h at 37 °C, the reaction system was measured spectrophotometrically at 562 nm to determine the content of the hydroxylated salicylate complex. Each sample was measured six times. The percentage scavenging effect was calculated by Equation (6):

HO• scavenging rate (%) = [1 – (A_1_ – A_2_)/A_0_] × 100% (6)

A_0_: Absorbance of the control; A_1_: Absorbance of the sample; A_2_: Absorbance of the reagent blank without sodium salicylate.

### 2.6. Statistical Analysis 

Unless otherwise indicated, each experiment was repeated three times, and the results are presented as mean ± standard deviation (± SD). Statistical analysis was performed using one-way analysis of variance (ANOVA) using SPSS 17.0, *p* < 0.05 was considered statistically significant. 

## 3. Results and Discussion

### 3.1. Preparation and Characterization of Astaxanthin Aggregate-Embedded Nano-Suspensions

**Figure 1:**
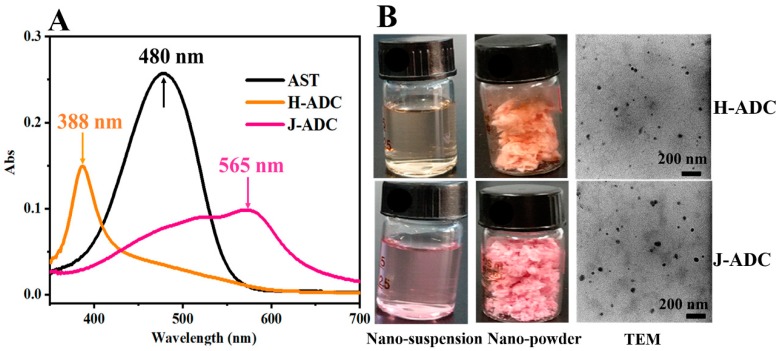
(**A**) Ultraviolet and visible spectrophotometry (UV-Vis) spectra of astaxanthin (AST), AST H-aggregates/DNA/chitosan nanocomplex (H-ADC) and AST J-aggregates/DNA/chitosan nanocomplex (J-ADC); (**B**) Pictures of H -ADC and J-ADC nano-suspensions, nano-powders, and their transmission electron microscopy (TEM) photos.

AST H- and J-aggregates are uncontrollable aggregation forms that only form in certain conditions, e.g., in hydrated solvents with a certain volume ratio. Here, two types of AST aggregates were incorporated into DNA/chitosan co-assemblies, and two different ADC nano-suspensions were successfully prepared. The unstable AST aggregates can be stabilized by incorporation into the hydrophobic micro-domain of the DNA/chitosan complex even without the presence of ethanol/water solvent. As the pattern of AST aggregation can be identified by the optical absorbance [31], the aggregation forms of AST that stabilized in the DNA/chitosan nano-system were evaluated by UV-Vis. As shown in (Figure 1A), the yellow nano-suspension (prepared by method 1) had an absorption peak at 388 nm, which was similar to that of the unstable H-aggregates, and it was 92 nm blue shifted relative to AST monomers [26]. This indicated the formation and encapsulation of AST H-aggregates in the DNA/chitosan nanoparticles, i.e., H-ADC. Comparatively, the absorption peak of the pink nano-suspension (prepared by method 2 and noted as J-ADC) was red shifted to 565 nm, accompanied by an acromion at 525 nm, which was similar to that of the unstable J-aggregates [32]. Both H-ADC and J-ADC were stable in water or in the dehydration form (Figure 1B), indicating that AST H- or J-aggregates can be stabilized in DNA/chitosan nanoparticles through intermolecular interaction between aggregates and certain molecular-skeletons (DNA or chitosan). The formation of two types of water-dispersible AST aggregates by using the same carrier materials is of significant merit, as AST H-aggregates and J-aggregates are not stable in solvents according to previous studies [33]. Although H- or J-aggregates were fabricated by carefully adjusting the ratio of ethanol/water as the solvent, the aggregates were unstable for long-term storage. With the help of DNA and chitosan, AST H and J-aggregates can be anchored in the hydrophobic micro-domain of the macromolecules during the ethanol evaporation process to form stable H-ADC and J-ADC nanoparticles.

During the preparation process, hydrophilic DNA and chitosan not only facilitated the formation and stabilization of AST aggregates, but also helped disperse hydrophobic AST aggregates in water without using any other solvent. According to the dynamic light scattering analysis (Table 1), the zeta potential of H-ADC and J-ADC was 34.1 and 36.5 mV, respectively. The positive charge of the nanoparticles could be attributed to the amino groups of chitosan present on the surface of the particles. Consequently, the positively charged H-ADC and J-ADC nanoparticles with the favorable membrane affinity of chitosan are anticipated to show extraordinary cellular affinity. The hydrodynamic size of H-ADC and J-ADC was 102.5 and 200.1 nm, respectively. The difference in size could be attributed to the spatial difference of closely stacked H-aggregates and loosely stacked J-aggregates. However, little difference in particle size between H-ADC and J-ADC was observed by TEM (Figure 1B), indicating their similar composition and size after dehydration. The encapsulation efficiency and loading capacity of AST in H-ADC was 99.14% and 8.69%, respectively, which was similar to that of J-ADC (Table 1). These results indicated that different types of ADC nano-suspensions share similar chemical compositions, which ensured the subsequent comparative analysis of the properties and functions of H-ADC and J-ADC.

**Figure 2:**
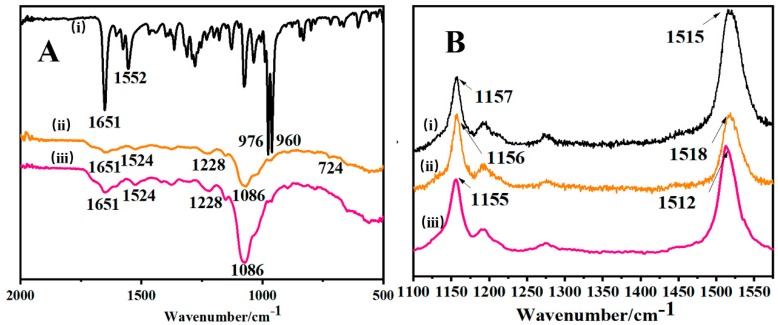
(**A**) Fourier transform infrared spectroscopy (FTIR) of AST (i), H-ADC (ii) or J-ADC (iii). (**B**) Raman spectroscopy analysis of AST (i), H-ADC (ii) or J-ADC (iii).

The FT-IR spectrum of pure AST, H-ADC and J-ADC are shown in Figure 2A. AST is a large polyene chain terminated with singly unsaturated chiral rings. Typical peaks of AST at 1651, 1552, 976 and 960 cm^−1^ were assigned to the C–O, C=C, and C–H stretching vibration peaks of the molecular skeleton, respectively. Compared with AST, the appearance of the vibration bands in H-ADC and J-ADC at 1086 and 1228 cm^−1^ for the symmetric and asymmetric stretching of the PO_2_ group, as well as the N–H bending vibration of the chitosan band at 1524 cm^−1^ well illustrated that H-ADC and J-ADC were constructed based on the non-covalent interactions among chitosan, DNA and AST [34]. Moreover, ADC nanoparticles showed considerably decreased peak intensities at 1651, 976 and 960 cm^−1^ relative to AST, accompanied with the disappearance of the C=C stretching vibration peak at 1552 cm^−1^, further indicating the embedment of AST in the inner-core of the DNA-chitosan nanocarriers due to the polar interactions between the host and guest molecules [35]. It is worth noting that a new small peak at 724 cm^−1^, which only existed in H-ADC, was assigned to out-of-plane bending modes of the weak hydrogen bonds to the OH groups. It has been reported that the formation of H-aggregates requires more hydrogen bonding, while J-aggregates can be formed only in the absence of hydrogen bonding [36]. Our results directly confirmed the earlier hypothesis that H-aggregates were conjugated together by hydrogen bonding. Because the formation of AST aggregates cannot significantly affect molecular vibrations, it remains challenging to distinguish the structural differences between H-ADC and J-ADC from FT-IR spectra. Therefore, more detailed information about H- or J-ADC was provided and analyzed by subsequent Raman spectroscopy [26,37].

Raman spectra can provide important information about high-energetic vibrations of assembled carotenoid aggregates [38]. Here, the Raman spectra of AST, H-ADC and J-ADC were evaluated to determine the changes in conjugated C=C bonds in polyene. As shown in Figure 2B, the most significant changes occurred in the tensile energy levels of C–C and C=C functional groups with Raman activity. According to the Raman spectrum of AST, the peaks at 1157 and 1515 cm^–1^ were attributed to the stretching vibrations of C–C and C=C bonds, respectively. In the spectrum of J-ADC, the C=C stretching mode (1512 cm^–1^) is red-shifted by about 3 cm^–1^ relative to that of AST (1515 cm^–1^), and the C–C stretching frequency was down-shifted (1155 cm^–1^) compared to AST (1157 cm^–1^). As for H-ADC, the peak position corresponding to the C–C stretching modes was red shifted by 1 cm^–1^, while the C=C stretching mode blue shifted by 3 cm^–1^ as compared with that of AST. Differences in C–C stretching vibrations could be related to the changed steric surroundings of methyl groups caused by planar AST molecules stacking, which is in accordance with previous reports [26]. The alterations of the C=C stretching frequencies after the formation of aggregates may be attributed to the alterations in the total molecular force field, as well as the geometry of AST in different aggregation forms by the non-covalent intermolecular interaction. These results showed that the Raman technique can effectively distinguish between these two AST aggregates.

### 3.2. Cytoprotective Effect of H-ADC and J-ADC Nanoparticles Against H_2_O_2_-Induced Oxidative Cell Damage in Caco-2 Cells

**Figure 3:**
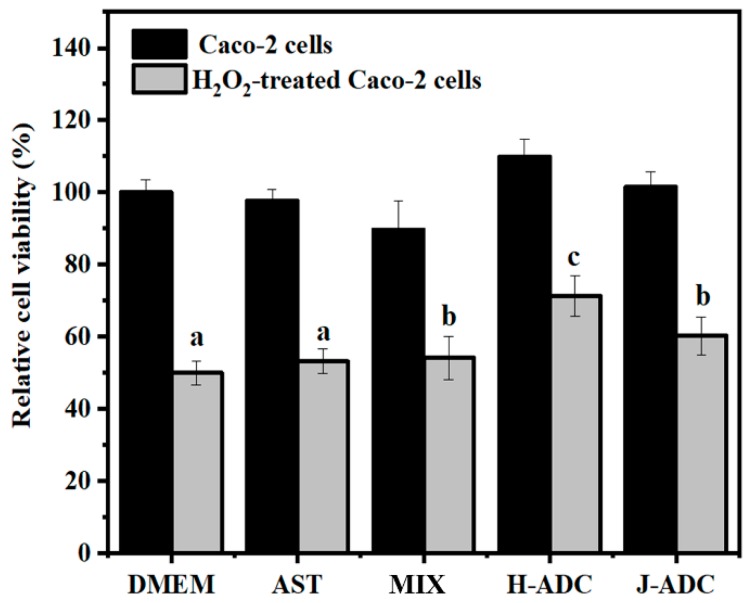
The cell viability of Caco-2 cells and H_2_O_2_-treated Caco-2 cells pretreated with different antioxidants (*n* = 6). a–c Different letters show statistically significant differences (*p* < 0.05) between different groups, whereas bars labelled with the same letter correspond to results that show no statistically significant differences.

Free radicals, such as ROS, are found to induce oxidative stress, DNA damage and cell death both in vitro and in vivo [39]. H_2_O_2_ and the active oxygen produced by its metabolism can easily penetrate the cell membrane and form highly active free radicals with iron ions in the cell [40]. Until now, the mechanism of cytoprotective effect of AST has not been clearly identified. However, it has been related to the antioxidant capacity of AST formulations, which acts against ROS and inactivates free radicals. As we know, AST tends to aggregate when present in lipid bilayers and other cellular environments [41]. Therefore, it is important to reveal the relationship between the aggregation patterns of AST and their possible roles in protecting cells from peroxidation damage. Here, H-ADC, J-ADC and a mixture of DNA, chitosan and AST with the same composition content (noted as MIX) were employed to evaluate their cytoprotective effect against Caco-2 cells under H_2_O_2_-induced oxidative stress. An identical amount of free AST was used as the positive control. According to the cytotoxicity assay, Caco-2 cells showed a survival rate of 50% after incubation with 5.8 mM of H_2_O_2_ for 1 h, noted as the model group (Figure 3). After pretreatment with antioxidants (H-ADC, J-ADC or AST), varying degrees of improvement were found in the cell viability of Caco-2 cells incubated with H_2_O_2_. Interestingly, H-ADC significantly improved the cell viability from 50% to 71%, while J-ADC only improved the cell viability to 60%. The enhanced cytoprotective effect of H-ADC is suggested to be caused by either the more tightly coupled H-aggregates of AST, by the smaller particle size of nanoparticles, or most likely, by a combined effect of both. It is worth mentioning that both H-ADC and J-ADC showed a higher cell viability rate than free AST (53%) and MIX (54%). One reason is that, regardless of the tightly packed H-aggregates or loosely coupled J-aggregates, the structure of AST aggregates guaranteed a relatively high local concentration of AST (with a loading capacity of approximately 8%) for efficient redox reactions. Another reason that led to the superior cytoprotective effect of ADC nanoparticles relative to free AST might be the efficient cellular uptake of the positively charged nanoparticles. These results indicated that the molecular aggregation state of AST played an important role in protecting cells from oxidative damage. In addition, H-ADC showed the strongest cytoprotective ability among the tested antioxidants, indicating the relationship between aggregation patterns and antioxidant functions of AST at the cellular level.

### 3.3. ROS Scavenging Efficiency of ADC Nanoparticles in Caco-2 Cells

**Figure 4:**
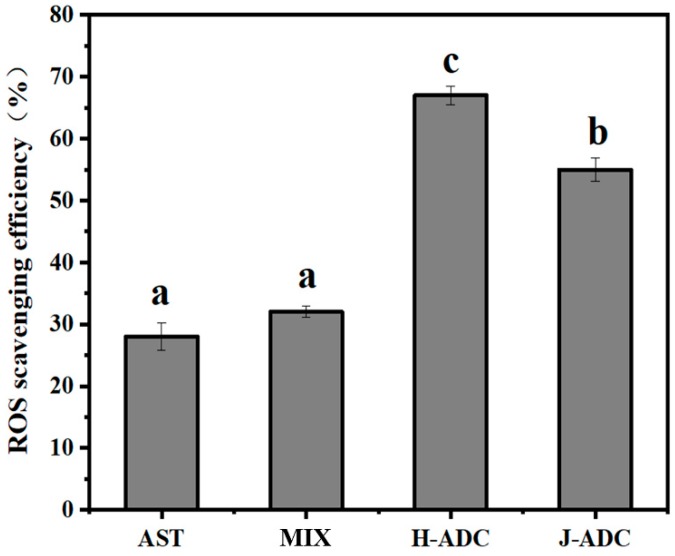
The ROS scavenging efficiency of different antioxidants in Caco-2 cells (*n* = 6). a–c Different letters show statistically significant differences (*p* < 0.05) between different groups, whereas bars labelled with the same letter correspond to results that show no statistically significant differences.

AST demonstrates significant antioxidant properties by efficiently interrupting the chain of oxidative reactions of free radicals like ROS. The presence of AST, such as the AST monomer, or assembled AST aggregates (H- or J-type), was found in various natural and artificial systems. Understanding the effects of the AST forms on their ROS eliminating ability is of great importance for their applications as free radical quenchers that alleviate oxidative stress injury and improve cell viability. Here, the ROS generation-suppressing effect of H-ADC, J-ADC, and a mixture of DNA, chitosan and AST with the same composition content (noted as MIX) was evaluated, respectively. Free AST with the same AST content was used as control. As shown in Figure 4, samples in AST monomer form (AST and MIX) showed a ROS scavenging efficiency of 28% and 32%, respectively, while samples in AST aggregate forms (H-ADC and J-ADC group) showed a relatively high scavenging efficiency of 67% and 55%, respectively. These results indicated that ROS elimination was mainly caused by AST, while biomacromolecules (DNA and chitosan) made little contribution to ROS scavenging. Interestingly, H-ADC showed the strongest ROS eliminating ability, with a ROS scavenging efficiency of 67%, more than twice that of equivalent free AST (28%) and 1.2-fold that of equivalent J-ADC. The difference might be attributed to the following factors: reactions like quenching ROS and dissipating the energy as heat, and scavenging ROS to prevent or terminate chain reactions, are concentration dependent [42]. The AST monomer tends to accumulate in cell membranes and protect cells from peroxidation [43]. However, due to the hydrophilic environment of the cytoplasm, the concentration of intracellular AST is quite low [44]. As for hydrophilic ADC nanoparticles, they are more easily internalized and accumulated in the cytoplasm. As a result, the intracellular chain of oxidative reactions is more easily prevented by ADC nanoparticles. Moreover, H-ADC possessed the H-type aggregation form of AST, which was compactly arranged with a high local density of AST molecules. Compared with loosely packed J-aggregates in J-ADC nanoparticles, higher local concentrations of AST and the smaller particle size of H-ADC are key factors that lead to better ROS scavenging efficiency at the cellular level.

### 3.4. Free Radicals Scavenging Rate of AST Aggregate Nanoparticles

**Figure 5:**
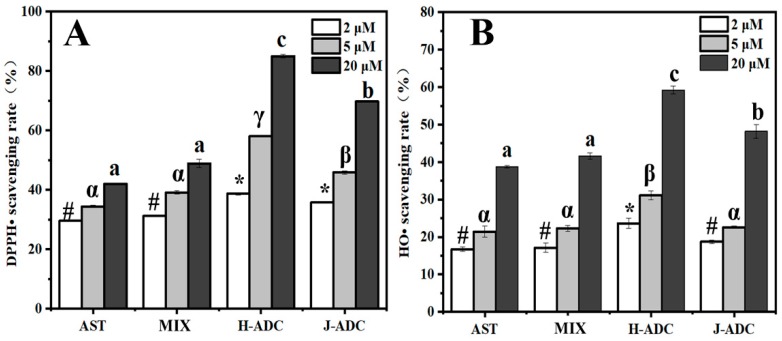
**(A)** DPPH• scavenging rate of different samples; (**B**) HO• scavenging rate of different samples. #, *, α–γ, a–d different letters and symbols show statistically significant differences (*p* < 0.05) between different groups, whereas bars labelled with the same letter correspond to results that show no statistically significant differences.

AST exerts its antioxidant ability by efficiently quenching dangerous free radicals. To directly prove the free radical scavenging ability of H-ADC and J-ADC, 1,1-diphenyl-2-picrylhydrazyl free radicals (DPPH•) and hydroxyl radicals (HO•) were produced through test tube experiments and used for scavenging rate analysis. As for DPPH• scavenging analysis (Figure 5A), the scavenging rate of each antioxidant sample increased along with the increase of AST concentration. When the concentration of AST was lower than 5 μM, the scavenging rate of each antioxidant still showed a significant difference (*p* < 0.05). However, AST aggregate-embedded nanoparticles (H-ADC and J-ADC) showed higher scavenging capacities than AST monomer-formed samples (AST and MIX) with an increasing concentration of AST. Notably, H-ADC with the AST concentration of 20 μM showed the highest scavenging rate (85%) compared to any other group with the same AST content. It is worth mentioning that nanoparticles containing H-aggregates (H-ADC, 85%) showed significantly higher scavenging rate than the nanoparticles containing J-aggregates (J-ADC, 69%), although the only difference in the two samples is the aggregation pattern of AST (*p* < 0.05). Similar results can also be found in hydroxyl radical-scavenging measurements in vitro. As shown in Figure 5B, the scavenging rate of H-ADC (62%) was 1.29-fold higher than that of J-ADC (48%) when the AST concentration was 20 μM. The excellent DPPH• and HO• scavenging effect of H-ADC indicated that H-ADC had superior antioxidant ability relative to J-ADC and free AST. It has been reported that H and J-type AST aggregates display unique optical, and structural properties, which were different from their monomer form [45]. Here, we confirmed the difference in antioxidant activity of various types of AST aggregates through in vitro evaluation for the first time as far as we know. 

It was speculated that the excellent antioxidant properties of AST was due to the cooperative effects of oxygen-containing groups, in which the ketone group activates the hydroxyl group, which resulted in the transfer of hydrogen atoms to peroxyl radicals [46]. Due to the newly formed intermolecular hydrogen bond, as well as the existence of the π-π conjugate structure in the intermolecular aggregation, different electron transport ability between H- and J-type aggregates could be found as a possible explanation for their different antioxidant activities [47]. Therefore, the antioxidant activity of AST is not only determined by its chiral structure, but also by its aggregation form.

## 4. Conclusions

In summary, two kinds of AST colloidal systems with stable AST H- and J-aggregates were prepared by using the same biomacromolecules (natural DNA and chitosan). The aggregation patterns of AST were mainly determined by the solvent effect and the initially-interacted molecule, which finally influenced the spectral characteristics and antioxidant activities of the colloidal system. In vitro comparative experiments on the antioxidant property indicated that, aggregated AST has better antioxidant activities than AST monomers with consistent compositions. Moreover, H-aggregates showed the best activity among the other AST aggregates. The results primarily confirmed our hypothesis that aggregation pattern influences the reactivity of bioactive compounds. The π-π conjugate structure of AST aggregates and intermolecular hydrogen bonding contribute to higher electron transport efficiency in H-type AST aggregates, and therefore lead to better antioxidant activity relative to J-aggregates. Our deduction that the aggregation form of AST influences its reactivity in oxidation-reduction reactions will contribute to the rational design and efficient utilization of AST.

## Figures and Tables

**Table 1:** Characterization of H-ADC and J-ADC.

Samples	Size (nm)	PdI	Zeta (mV)	LC ^1^ (%)	EE ^2^ (%)
H-ADC	102.5 ± 0.3 ^a^	0.31 ± 0.02 ^a^	34.1 ± 0.4 ^a^	8.69 ± 0.02 ^a^	99.14 ± 0.03 ^a^
J-ADC	200.1 ± 0.8 ^b^	0.33 ± 0.02 ^a^	36.5 ± 0.7 ^a^	8.71 ± 0.01 ^a^	99.16 ± 0.07 ^a^

^1^ Loading capacity; ^2^ Encapsulation efficiency; ^a,b^ Different letters show statistically significant differences between the H-ADC and J-ADC (*p* < 0.05).

## Abbreviations

AST	astaxanthin
DMEM	Dulbecco’s modified Eagle medium
DCFH-DA	2,7-dichlorodihydrofluorescin diacetate
DPPH•	1,1-diphenyl-2-picrylhydrazyl free radical
FITC	fluorescein isothiocyanate
FT-IR	Fourier transform infrared spectroscopy
HO•	hydroxyl free radical
H-ADC	AST H-aggregates/DNA/chitosan nanocomplex
J-ADC	AST J-aggregates/DNA/chitosan nanocomplex
MTT	3-(4,5-dimethylthiazol-2-yl)-2,5-diphenyl-tetrazolium bromide
ROS	reactive oxygen species
TEM	transmission electron microscopy
UV-Vis	ultraviolet and visible spectrophotometry
